# Role of NKCC1 Activity in Glioma K^+^ Homeostasis and Cell Growth: New Insights With the Bumetanide-Derivative STS66

**DOI:** 10.3389/fphys.2020.00911

**Published:** 2020-07-31

**Authors:** Lanxin Luo, Jun Wang, Dawei Ding, Md Nabiul Hasan, Sung-Sen Yang, Shih-Hua Lin, Philipp Schreppel, Baoshan Sun, Yan Yin, Thomas Erker, Dandan Sun

**Affiliations:** ^1^ Department of Neurology, University of Pittsburgh, Pittsburgh, PA, United States; ^2^ Department of Neurosurgery, University of Minnesota, Minneapolis, MN, United States; ^3^ Department of Neurology, The Second Hospital of Dalian Medical University, Dalian, China; ^4^ Division of Nephrology, Department of Medicine, Tri-Service General Hospital, National Defense Medical Center, Taipei, Taiwan; ^5^ Department of Medicinal Chemistry, University of Vienna, Vienna, Austria; ^6^ Pólo Dois Portos, Instituto National de Investigação Agrária e Veterinária, I.P., Quinta da Almoinha, Dois Portos, Portugal; ^7^ Veterans Affairs Pittsburgh Health Care System, Geriatric Research, Educational and Clinical Center, Pittsburgh, PA, United States

**Keywords:** bumetanide, bumetanide-derivative STS66, cell volume regulation, glioma, K^+^ influx, Na^+^-K^+^-Cl^−^ cotransporter

## Abstract

**Introduction:** Na^+^-K^+^-2Cl^−^ cotransporter isoform 1 (NKCC1) is important in regulating intracellular K^+^ and Cl^−^ homeostasis and cell volume. In this study, we investigated a role of NKCC1 in regulating glioma K^+^ influx and proliferation in response to apoptosis inducing chemotherapeutic drug temozolomide (TMZ). The efficacy of a new bumetanide (BMT)-derivative NKCC1 inhibitor STS66 [3-(butylamino)-2-phenoxy-5-[(2, 2, 2-trifluoroethylamino) methyl] benzenesulfonamide] in blocking NKCC1 activity was compared with well-established NKCC1 inhibitor BMT.

**Methods:** NKCC1 activity in cultured mouse GL26 and SB28-GFP glioma cells was measured by Rb^+^ (K^+^) influx. The WNK1-SPAK/OSR1-NKCC1 signaling and AKT/ERK-mTOR signaling protein expression and activation were assessed by immunoblotting. Cell growth was determined by bromodeoxyuridine (BrdU) incorporation assay, MTT proliferation assay, and cell cycle analysis. Impact of STS66 and BMT on cell Rb^+^ influx and growth was measured in glioma cells treated with or without TMZ.

**Results:** Rb^+^ influx assay showed that 10 μM BMT markedly decreased the total Rb^+^ influx and no additional inhibition detected at >10 μM BMT. In contrast, the maximum effects of STS66 on Rb^+^ influx inhibition were at 40–60 μM. Both BMT and STS66 reduced TMZ-mediated NKCC1 activation and protein upregulation. Glioma cell growth can be reduced by STS66. The most robust inhibition of glioma growth, cell cycle, and AKT/ERK signaling was achieved by the TMZ + STS66 treatment.

**Conclusion:** The new BMT-derivative NKCC1 inhibitor STS66 is more effective than BMT in reducing glioma cell growth in part by inhibiting NKCC1-mediated K^+^ influx. TMZ + STS66 combination treatment reduces glioma cell growth *via* inhibiting cell cycle and AKT-ERK signaling.

## Introduction

Ion homeostasis and cell volume regulation play an important role in many cellular functions, including intracellular metabolism, transepithelial transport, cell migration, cell growth, and cell death (Wehner et al., 2003; Delpire and Gagnon, 2018). Intracellular K^+^ concentration in most cells is ~140–150 mM, which is established by Na/K ATPase-mediated K^+^ influx and K^+^ channels-mediated K^+^ influx or efflux to maintain optimal physiological function and cell volume (Thier, 1986; Skou and Esmann, 1992). At physiological concentrations, intracellular K^+^ ion homeostasis is an important regulator of caspase and apoptotic nuclease function (Hughes and Cidlowski, 1999). One of the first events at the onset of apoptosis is cell shrinkage [apoptotic volume decrease (AVD)], resulting from loss of intracellular K^+^ and Cl^−^ and cell volume (Okada and Maeno, 2001; Bortner and Cidlowski, 2007). A huge loss of cell K^+^ serve as a apoptosis signal allowing execution of the apoptosis program by cytochrome c release, caspase-3 activation, and endonuclease activation (Okada and Maeno, 2001; Yu, 2003). Cell shrinkage is usually followed by activate cell volume regulatory ion transporters Na^+^/H^+^ exchanges (NHE), Na^+^-K^+^-2Cl^−^ cotransporter (NKCC1), Cl^−^/HCO_3_^−^ anion exchanges (AE), and non-selective cation channels to regulatory volume increase (RVI; Maeno et al., 2006; Hoffmann et al., 2009), which counteracts AVD and thereby apoptosis (Hoffmann and Lambert, 2014). In addition, early studies strongly suggest that K^+^ and Cl^−^ ion channels and ion transporters are associated to chemoresistance (Poulsen et al., 2010; Algharabil et al., 2012) and present as new targets for anti-tumor therapy.

NKCC1 belongs to the *SLC12A* family of cation-chloride cotransporters (Gamba, 2005) and plays an important role in intracellular K^+^, Cl^−^ accumulation and RVI in response to osmotic stress or AVD (Hoffmann et al., 2009; Algharabil et al., 2012; Delpire and Gagnon, 2018). NKCC1 protein expression was higher in human glioma cells than in normal control cortex and localized at the leading edge of human glioma cells (Aronica et al., 2007; Haas and Sontheimer, 2010; Garzon-Muvdi et al., 2012; Schiapparelli et al., 2017). Moreover, NKCC1 protein expression has been shown to associate with glioma cell migration (Zhu et al., 2014) *via* regulation of focal adhesion dynamics, cell contractility, and cell volume (Haas et al., 2011; Garzon-Muvdi et al., 2012). We have reported recently that temozolomide (TMZ) monotherapy significantly upregulated NKCC1 protein expression and activity (NKCC1-mediated Rb^+^ influx; Luo et al., 2020) to replenish intracellular K^+^ in response to TMZ induced-apoptosis. NKCC1 inhibitor bumetanide (BMT) in combination with TMZ accelerated apoptosis, reduced tumor volume, and potentiated the cytotoxic effects of TMZ in the GL26 and SB28-GFP intracranial mouse syngeneic glioma model (Luo et al., 2020).

In this study, using two different glioma cell lines (GL26 and SB28-GFP), we further investigated the efficacy of a new BMT-derivative NKCC1 inhibitor STS66 along with well-established NKCC1 inhibitor BMT on regulating glioma NKCC1 activity, K^+^ influx, and cell growth in response to TMZ. STS66 significantly reduced TMZ-induced NKCC1 activation and glioma cell growth compared to BMT.

## Materials and Methods

### Materials

BMT (#B3023), TMZ (#T2577), propidium iodide (PI, #P4864), and MTT (#M2128) were purchased from Sigma-Aldrich (St. Louis, MO). Dulbecco’s Modified Eagle Medium (DMEM/HEPES, Cat# 12430-054) and Penicillin/streptavidin (Cat# 15240062) were from Gibco (Carlsbad, CA). Fetal bovine serum (FBS) was obtained from invitrogen (Carlsbad, CA). Anti-phospho-NKCC1(pThr206) antibody, anti-phospho-SPAK/OSR1 (pSer383 SPAK/pSer325 OSR1) antibody, and anti-total-SPAK/OSR1 (tSPAK/tOSR1) antibody were developed by Dr. Yang (Taiwan National University) and validated in previous studies (Moriguchi et al., 2005; Yang et al., 2010). Monoclonal anti-total NKCC was from the Developmental Studies Hybridoma Bank (T4, Iowa City, IA). Antibody against α-tubulin (Cat #2125), rabbit anti-phospho AKT (Ser473; Cat# 9271), rabbit anti-AKT (Cat# 4691), rabbit anti-phospho ERK (Thr202/Tyr204; Cat# 4370), rabbit anti-ERK (Cat# 4695), and rabbit anti-phospho p70 S6k (T389; Cat# 9234) were from cell signaling (Beverly, MA). Mouse anti-p70 S6K (Cat# sc-8418) was purchased from Santa Cruz Biotechnology (Dallas, TX). BCA Protein Assay Kit (Cat #23227) was from Thermo Scientific (Rockford, IL). STS66 was synthesized by Töllner et al. (2014) as described previously.

### Cell Cultures and Authentication

Immunogenic mouse glioma GL26 and non-immunogenic mouse SB28-GFP glioma cells were used as previously described (Kohanbash et al., 2017). GL26 glioma cell line was obtained from Prof. Vadlamudi of University of Texas Health, San Antonio (Sareddy et al., 2016). Glioma cells were maintained in DMEM/HEPES containing 10% heat-inactivated FBS, 2 mM L-glutamine, 1x penicillin/streptavidin, and 1 mM sodium pyruvate. Cultures were passaged approximately every 4 days with fresh medium at a density of 10^6^ cells/75 cm^2^ in a culture flask. Passage 8–30 of glioma cells were used in the study. All cell lines were authenticated by short tandem repeat (STR) DNA finger printing (by IDEXX BioResearch, Columbia, MO). In addition, PCR analysis was performed to confirm the absence of mycoplasma infection in all cell cultures.

### NKCC1-Mediated Rubidium (Rb^+^) Influx Assay

GL26 or SB28-GFP cells seeded in 24-well plates were exposed to either isotonic (310 mOsm) or hypertonic (400 mOsm) solutions containing different concentrations of BMT (0, 10, 20, 40, and 60 μM) or STS66 (0, 10, 20, 40, and 60 μM). Rb^+^ influx into cells under above conditions was assayed. Briefly, to measure Rb^+^ uptake in glioma cells, the culture medium was removed and cells were rinsed with an isotonic wash buffer (310 mOsm, containing 134 mM NaCl, 2 mM CaCl_2_, 0.8 mM NaH_2_PO_4_, 5 mM glucose, 25 mM HEPES, and 1.66 mM MgSO_4_). Cells were exposed to the isotonic buffer containing 5.36 mM Rb^+^ in the absence or presence of BMT or STS66 (0–60 μM) for 10 min at 37°C. To measure Rb^+^ influx in glioma cells in response to hypertonic stress, cells were exposed to the hypertonic solution (400 mOsm adjusted with 7.7 mM sucrose, 5.36 mM Rb^+^) for 5 min at 37°C. To terminate Rb^+^ influx, cells were washed with the isotonic or hypertonic washing solutions (Rb^+^ free) and lysed with 0.15% SDS (200 μl/well) to release intracellular Rb^+^. The intracellular Rb^+^ concentration in cell lysates was measured using an automated atomic absorption spectrophotometer (Ion Channel Reader, ICR-8000; Aurora Biomed, Vancouver, Canada). Total protein of cell lysates was measured by BCA assay. NKCC1-mediated Rb^+^ influx was determined by subtracting Rb^+^ influx value in the presence of BMT or STS66 from one in the absence of the drug. Rb^+^ influx rate was calculated and presented as μg Rb^+^/mg protein/min. For the chronic drug treatment cultures, GL26 and SB28-GFP cells seeded in 24-well plates (60% confluent) were incubated with vehicle (Veh, 0.1% DMSO in PBS), TMZ (100 μM), BMT (10 μM), STS66 (60 μM), TMZ+ BMT or TMZ + STS66 in the culture medium for 48 h. Rb^+^ influx in these cells was determined as described above.

### Immunoblotting

GL26 and SB28-GFP cells were washed with ice-cold PBS and incubated in RIPA buffer containing one pill of phosSTOP and 2 mM protease inhibitors as described before (Algharabil et al., 2012). Cells were lysed by sonication at 4°C. Protein content of the cellular lysate was determined with BCA assay. Samples in the sample buffer (Thermo Scientific, Rockford, IL, USA) were boiled at 95°C for 5 min. The samples were then electrophoretically separated on 4–15% SDS gels. After transferring to polyvinylidene difluoride (PVDF) membranes, the blots were blocked in 10% nonfat dry milk in Tris-buffered saline-T (TBS-T, 0.05% Tween-20) for 1 h at room temperature and then incubated with appropriate primary antibodies (p-NKCC1, 1:300; t-NKCC1, 1:2000; p-WNK1, 1:1000; t-WNK, 1:500; p-SPAK/pOSR1, 1:500; t-SPAK/OSR1, 1:500; p-AKT, 1:500; t-AKT, 1:1000; p-ERK, 1:1000; t-ERK, 1:1000; p-p70 S6K, 1:1000; p70 S6K, 1:1000) at 4°C overnight. After rinsing with TBS-T, the blots were incubated with horseradish peroxidase-conjugated secondary IgG (1:2000) for 1 h at RT. Bound antibody was visualized with an enhanced chemiluminescence assay. Protein band signal intensities were analyzed using ImageJ and normalized to α-tubulin expression.

### BrdU Incorporation Assay

Cell proliferation of GL26 and SB28-GFP cells was measured by quantifying bromodeoxyuridine (BrdU) incorporation. Cells were treated with DMSO vehicle (Con-Veh), TMZ (T, 100 μM), BMT (B, 10 μM), STS66 (S, 10 μM or 60 μM), or T + B, T + S for 24 or 48 h. BrdU was added in the last 4 h period of 24 or 48 h incubation. The incorporation of BrdU into newly synthesized DNA of proliferating cells was detected by using a peroxidase-conjugated antibody, which reacts with the thymidine analogue BrdU. Bound anti-BrdU-peroxidase conjugated antibody was measured by a substrate reaction, and then quantified calorimetrically by a microplate reader (Molecular Devices, Sunnyvale, CA) at dual wavelength of 450/550 nm.

### MTT Proliferation Assay

Cells were treated with Con-Veh, TMZ (T, 100 μM), BMT (B, 10 μM), STS66 (S, 10 μM or 60 μM), or T + B, T + S for 48 h. The media of each well of a 96-well plate were replaced with 100 μl of fresh media containing 0.5 mg/mL MTT (Thiazolyl Blue Tetrazolium Bromide) solution. After 4 h incubation, the dark blue water insoluble MTT-formazan crystals were dissolved in 100 μl DMSO and the absorbance of each well was determined at 570 nm with a microplate reader (Molecular Devices, Sunnyvale, CA).

### Cell Cycle Analysis

Cell cycle analysis was performed as previously described (Algharabil et al., 2012). After staining the DNA with PI, data were acquired in a BD LSRII instrument. Cell cycle distribution was calculated with Flow Jo (Tree Star) software.

### Statistical Analysis

The results were expressed as the mean ± standard error of the mean (SEM). *N* values represent the number of independent experiments. Statistical significance was determined using one-way analysis of variance by GraphPad Prism 8 (GraphPad Software, Inc.). One-way and two-way ANOVA were conducted for multiple comparisons. Data are significant when a value of *p* was *p* < 0.05.

## Results

### NKCC1-Mediated Rb^+^ Influx in Cultured Glioma Cells and in Response to BMT or STS66

**Figure 1:**
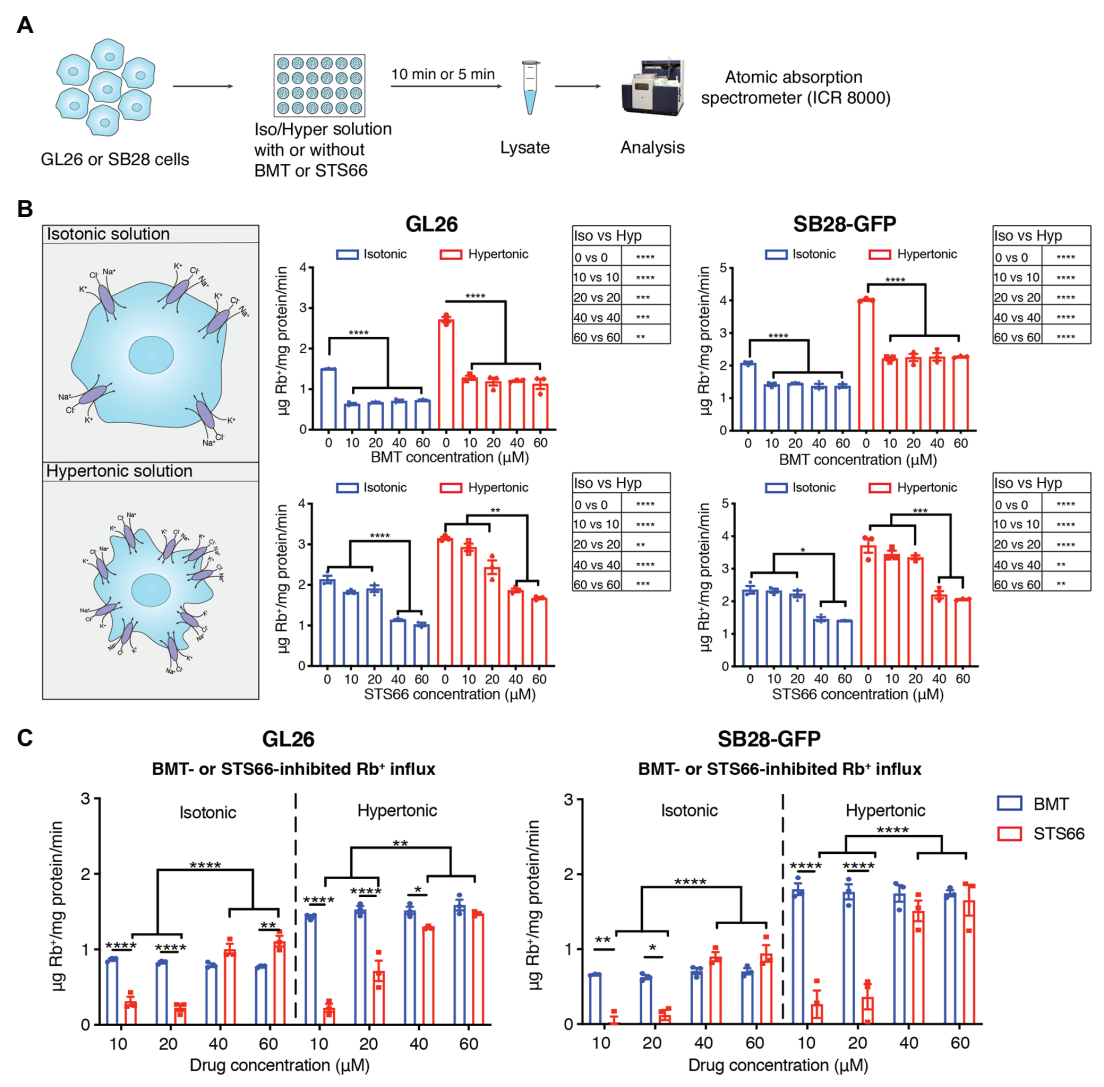
STS66 reduces NKCC1-mediated Rb^+^ influx in cultured glioma cells in a dose-dependent manner. **(A)** Experimental protocol. **(B)** Total Rb^+^ influx. GL26 or SB28-GFP cells were exposed to either isotonic (310 mOsm) or hypertonic (400 mOsm) solutions containing different concentrations of bumetanide (BMT; 0, 10, 20, 40, and 60 μM) or STS66 (0, 10, 20, 40, and 60 μM). Rb^+^ influx into cells under above conditions was assayed and determined using ICR 8000. **(C)** BMT- or STS66-inhibited Rb^+^ influx (NKCC1 activity). Data are means ± SEM (*n* = 3), ^*^p < 0.05, ^**^p < 0.01, ^***^p < 0.001, ^****^p < 0.0001.

We first assessed total K^+^ (Rb^+^) influx in GL26 and SB28-GFP glioma cells under isotonic conditions or hypertonic osmotic stress when NKCC1 activity is stimulated with ICR8000 (Figure 1A). BMT- or STS66-inhibited Rb^+^ uptake was determined as an index for NKCC1 activity. As shown in Figure 1B, exposure of GL26 glioma cells to 10–60 μM BMT decreased the total Rb^+^ uptake by ~54.3% (*p* < 0.0001) in isotonic conditions (310 mOsm). No additional inhibition was observed with BMT at >10 μM (*p* > 0.05, Figure 1B), which is consistent with report showing that BMT (IC_50_ of 0.1 μM) with concentrations from 1 to 10 μM completely blocked NKCC1 activity (Brandt et al., 2018). In response to hypertonic osmotic stress (400 mOsm), GL26 glioma cells elevated the total Rb^+^ uptake by ~81.0% (*p* < 0.0001, Figure 1B). About 10–60 μM BMT blocked the hypertonic osmotic stress-induced Rb^+^ uptake by ~55.8% (*p* < 0.0001, Figure 1B), and the residual Rb^+^ influx remains higher than the level in isotonic conditions. Similar patterns in changes of total Rb^+^ influx were detected in SB28-GFP glioma cells with BMT. In the case of STS66, the total Rb^+^ influx either in GL26 or SB28-GFP glioma cells was not significantly decreased with 10 or 20 μM STS66 (*p* > 0.05, Figure 1B). However, 40 μM STS66 significantly inhibited the total Rb^+^ influx (*p* < 0.05) and no additional inhibition was detected when STS66 concentration increased to 60 μM (*p* > 0.05, Figure 1B). STS66 (at 40–60 μM) shows similar efficacy in inhibiting the total Rb^+^ influx under hypertonic osmotic stress conditions (Figure 1B). Analysis of BMT- or STS66-inhibited Rb^+^ influx (reflecting NKCC1 activity) was shown in Figure 1C. BMT (10–60 μM) showed similar Rb^+^ influx inhibition under either isotonic or osmotic shrinkage conditions. STS66-mediated inhibition of NKCC1 activity in GL26 and SB28-GFP cells more effective at 40–60 μM. These findings concluded that NKCC1 activity plays an important role in K^+^ influx in glioma cells, which are sensitive to both BMT- and STS66-mediated inhibition.

### STS66 Reduces Glioma K^+^ (Rb^+^) Influx in Response to Chemoreagent Temozolomide

**Figure 2:**
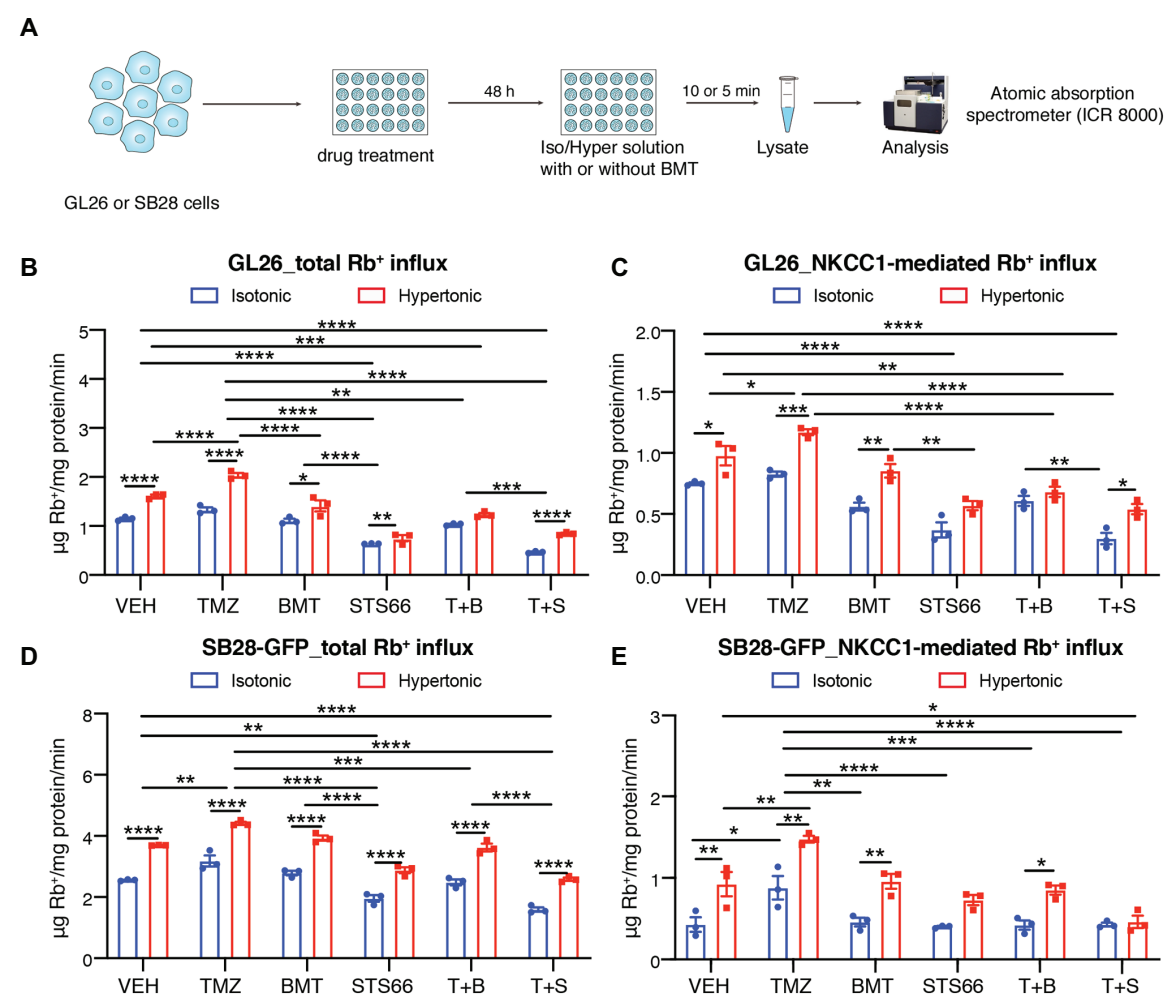
STS66 reduces glioma K^+^ (Rb^+^) influx in combination of chemoreagent temozolomide. **(A)** Experimental protocol. **(B–E)** Total Rb^+^ influx **(B,D)** and NKCC1-mediated Rb^+^ influx **(C,E)**. GL26 cells were exposed to DMSO (Veh), temozolomide (TMZ, 100 μM), BMT (10 μM), STS66 (60 μM), T + B, or T + S combined for 48 h. Rb^+^ influx into cells under either isotonic (310 mOsm) or hypertonic (400 mOsm) solutions was assayed for 5–10 min and determined using ICR 8000. Data are means ± SEM (*n* = 3), ^*^p < 0.05, ^**^p < 0.01, ^***^p < 0.001, ^****^p < 0.0001.

In response to chemo reagent TMZ-mediated AVD, glioma cells upregulate NKCC1 protein to counteract against loss of intracellular K^+^ and Cl^−^ and AVD (Algharabil et al., 2012; Zhu et al., 2014). Exposure of GL26 or SB28-GFP cells to TMZ for 48 h stimulated the total Rb^+^ uptake under isotonic conditions (~15.9 or 24.3%, respectively, *p* < 0.01, Figures 2A,B,D). The NKCC1-mediated Rb^+^ influx (determined in the presence of 10 μM BMT during Rb^+^ assay) was significantly increased in GL26 or SB28-GFP cells by TMZ (*p* < 0.05, Figures 2C,E). We further assessed whether chronic blockade of NKCC1 activity with BMT or STS66 alone or in combination with TMZ could alter glioma Rb^+^ influx, as an anti-tumor mechanism. Figures 2B,C show that among all treatment regimens, chronic exposure of glioma cells to STS66 is most effective in decreasing the total Rb^+^ as well as NKCC1-mediated Rb^+^ influx (~44.4 and 50.6%, respectively) under isotonic conditions (*p* < 0.0001) or under hypertonic conditions in GL26 cells (by ~53.5 and 41.7%; *p* < 0.0001). Moreover, the TMZ + STS66 combinatory treatment significantly reduced the NKCC1-mediated Rb^+^ uptake by ~60.0% in isotonic conditions (*p* < 0.0001) and by ~44.8% in hypertonic conditions in GL26 cells (*p* < 0.0001, Figure 2C). However, they are not significantly different from the STS66 monotreatment group. In comparison, the TMZ + BMT displayed reduced NKCC1-mediated Rb^+^ uptake under isotonic condition or hypertonic conditions (*p* < 0.0001, Figure 2C) but the effect is smaller than the STS66 monotreatment or the TMZ + STS66 combinatory treatment. SB28-GFP cells exhibited similar changes in Rb^+^ uptake under above conditions (Figures 2D,E). Taken together, these findings clearly demonstrate that STS66 is more effective than BMT in reducing NKCC1-mediated Rb^+^ influx in response to TMZ stimulation.

### STS66 Reduces Glioma Cell Proliferation and Induces G0/G1 Arrests in Combination With Chemoreagent Temozolomide

**Figure 3:**
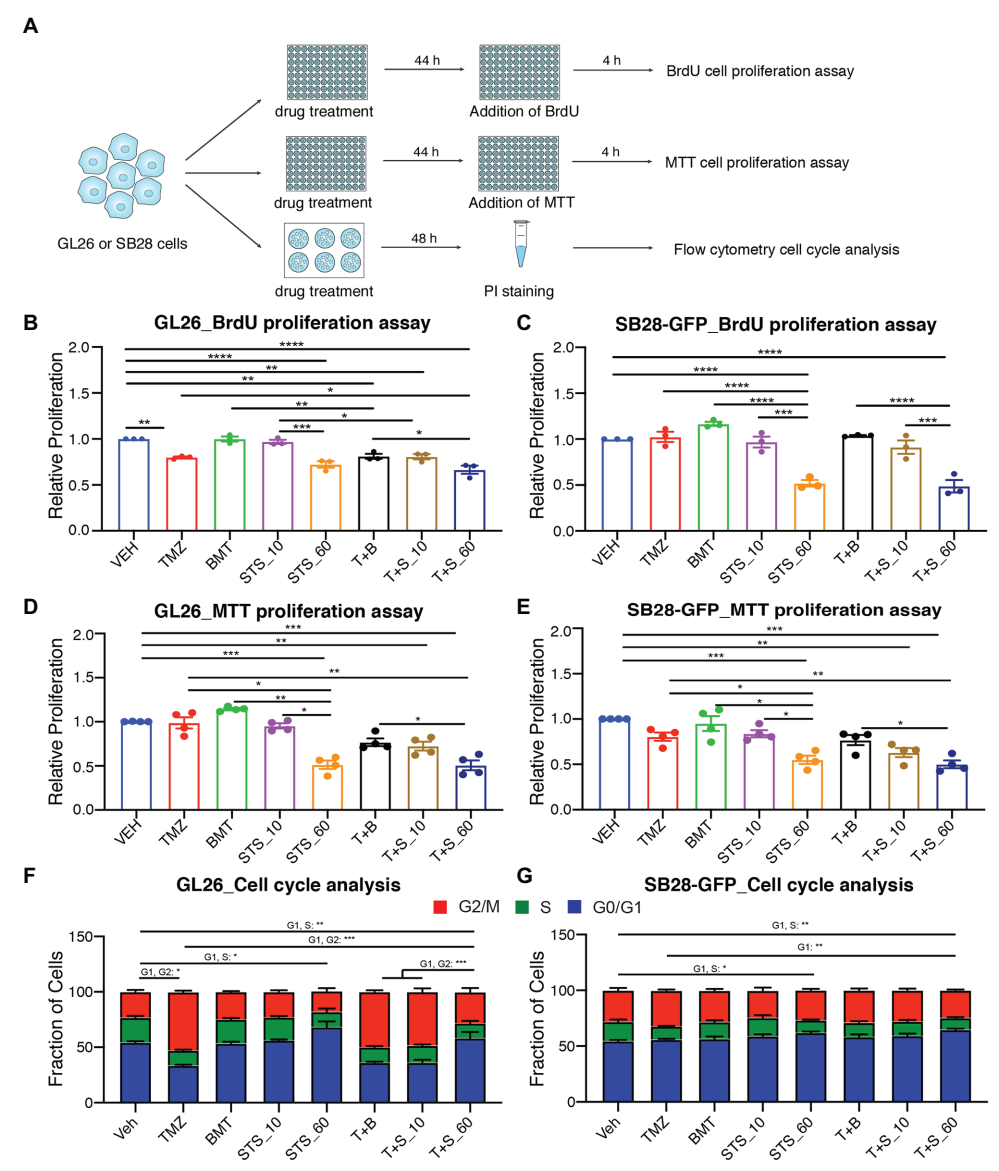
STS66 reduces glioma proliferation and cell cycle in combination of chemoreagent temozolomide. **(A)** Experimental protocol. GL26 or SB28-GFP cells were exposed to DMSO (Veh), TMZ (100 μM), BMT (10 μM), STS66 (10 and 60 μM), T + B, or T + S combined for 48 h and proliferation was determined by BrdU incorporation assay and MTT assay. Cell cycle distribution was analyzed by flow cytometry. **(B,C)** Relative cell proliferation determined by BrdU incorporation assay. Data are means ± SEM (*n* = 3), ^*^p < 0.05, ^**^p < 0.01, ^***^p < 0.001, ^****^p < 0.0001. **(D,E)** Relative cell proliferation determined by MTT assay. Data are means ± SEM (*n* = 4), ^*^p < 0.05, ^**^p < 0.01, ^***^p < 0.001. **(F,G)** Cell cycle distribution was analyzed by flow cytometry after DNA staining with propidium iodide (PI). Data are means ± SEM (*n* = 4), ^*^p < 0.05, ^**^p < 0.01, ^***^p < 0.001.

We subsequently investigated role of NKCC1 in glioma cell proliferation and cell cycle in response to chronic inhibition of NKCC1 with BMT, STS66, or in combination with TMZ (Figure 3A). Figures 3B,C show that TMZ treatment for 48 h displayed inhibited BrdU incorporation by in GL26 cells (*p* < 0.01) but not in SB28-GFP cells. Chronic exposure of glioma cells to BMT or STS66 (10 μM) alone or in combination treatment for 48 h did not inhibit GL26 or SB28-GFP cell proliferation. However, BrdU incorporation in GL26 and SB28-GFP cells was significantly inhibited after STS66 (60 μM) alone or in combination with TMZ. There were no significant differences between the two groups, STS66 alone and the T + S combination (Figures 3B,C). The similar pattern was found in GL26 and SB28-GFP cells with STS66 treated for 24 h (Supplementary Figure S1). These findings were further confirmed by MTT proliferation assay. Single-agent treatment for 48 h with TMZ, BMT, or STS66 (10 μM) did not change the proliferation of either GL26 or SB28 cell lines. In contrast, STS66 (60 μM) significantly reduced the proliferation of both GL26 and SB28 cells (*p* < 0.001, Figures 3D,E). The maximum inhibition effects were obtained with TMZ + STS66 (60 μM) combination (*p* < 0.001, Figures 3D,E). We next performed cell cycle analysis of GL26 and SB28-GFP cells treated with the above regimens for 48 h. In SB28-GFP cells, exposure of glioma cells to BMT or STS66 (10 μM) alone did not change the fraction of cells in any cell cycle state. In both GL26 and SB28 glioma cells, STS66 (60 μM) significantly increased the fraction of cells in G0/G1 phase and reduced in the S-phase of the cell cycle (*p* < 0.05, Figures 3F,G). TMZ + STS66 (60 μM) combination increased the G0/G1 fraction with a corresponding decrease of those in S-phase of the cell cycle (*p* < 0.01, Figures 3F,G). In summary, STS66 alone or in combination with TMZ reduced glioma cell proliferation and caused G0/G1 phase arrest.

### TMZ-Mediated Activation of WNK1-SPAK/OSR1-NKCC1 Signaling in Cultured Glioma Cells

**Figure 4:**
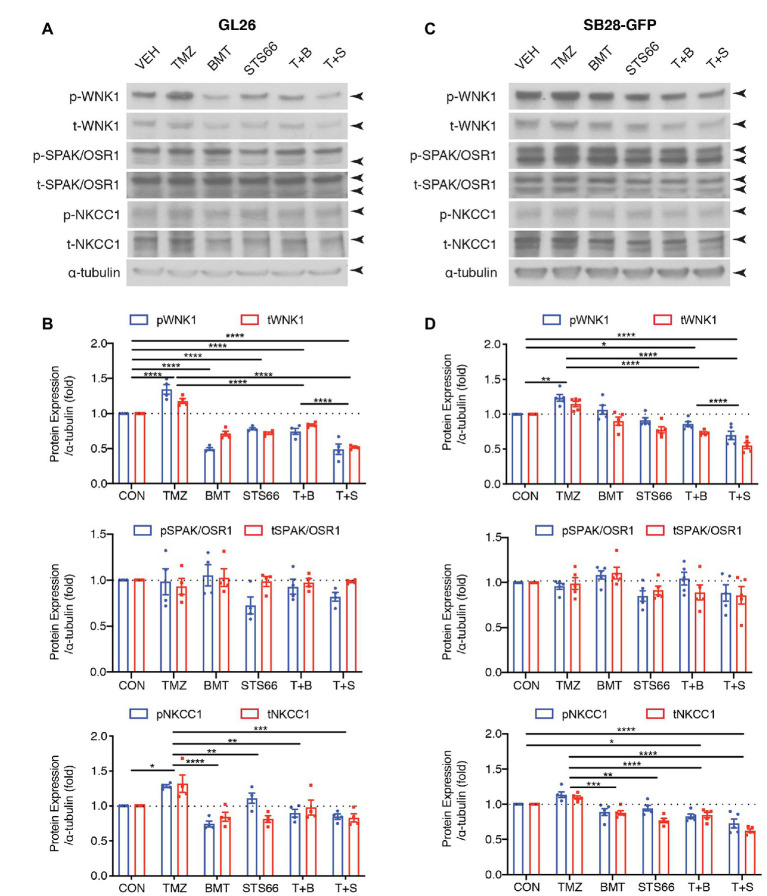
STS66 reduced activation of WNK1-SPAK/OSR1-NKCC1 cascade in cultured glioma cells in response to TMZ. **(A)** Representative immunoblotting images of total (t-) or phosphorylated (p-) WNK1, SPAK/OSR1, and NKCC1. GL26 cells were exposed to DMSO (Veh), TMZ (100 μM), BMT (10 μM), STS66 (10 μM), T + B, or T + S combined for 48 h. Cell lysates were harvested for immunoblotting. **(B)** Summary. Data are means ± SEM (*n* = 4, 5), ^*^p < 0.05, ^**^p < 0.01, ^***^p < 0.001, ^****^p < 0.0001. **(C)** Representative immunoblotting images of total (t-) or phosphorylated (p-) WNK1, SPAK/OSR1, and NKCC1. SB28-GFP cells were exposed to DMSO (Veh), TMZ (100 μM), BMT (10 μM), STS66 (10 μM), T + B, or T + S combined for 48 h. **(D)** Summary. Data are means ± SEM (*n* = 4, 5), ^*^p < 0.05, ^**^p < 0.01, ^***^p < 0.001, ^****^p < 0.0001.

We previously found that TMZ stimulated NKCC1 protein upregulation and activation in human primary glioma cells (GC#99 and GC#22) through With-No-K (lysine) kinase 1 (WNK1) and oxidative stress-responsive kinase 1 (OSR1) signaling pathway (Zhu et al., 2014). In this study, we further examined effects of chronic inhibition of NKCC1 activation *via* BMT or STS66 on WNK1-SPAK/OSR1-NKCC1 signaling pathway in glioma cells in response to TMZ. As shown in Figures 4A,B, exposing GL26 to TMZ for 48 h triggered an increase of total (t)-WNK1 expression by ~17.8% of control (*p* < 0.0001) and phosphorylated (p)-WNK1 expression by ~34.5% of control (*p* < 0.0001). Either p- or t-SPAK/OSR1 in GL26 were not significantly altered by TMZ (Figures 4A,B). Moreover, the combined treatment of TMZ + BMT or TMZ + STS66 significantly downregulated WNK1 protein (*p* < 0.0001). Incubation of GL26 glioma cells with TMZ for 48 h also triggered an increase of p-NKCC1 protein expression by ~28.3% and t-NKCC1 protein by ~32.0%, compared to Veh controls (Figures 4A,B). In contrast, exposing GL26 cultures to NKCC1 inhibitor BMT or STS66 in combination with TMZ significantly decreased TMZ-triggered upregulation of t-NKCC1 (*p* < 0.01) or p-NKCC1 (*p* < 0.0001; Figures 4A,B). The same pattern was also identified in SB28-GFP cells (Figures 4C,D). The ratios of p-/t-WNK1, p-/t-SPAK/OSR1, and p-/t-NKCC1 were presented in Supplementary Figure S2. The results show that BMT significantly reduced the ratio of p-/t-WNK1 in GL26 cells (Supplementary Figure S2A), mainly due to inhibition of p-WNK1 protein expression. No differences in the ratio of p-/t-SPAK/OSR1 or p-/t-NKCC1 were detected in different treatments (Supplementary Figures S2B–F) because that both phosphorylated and non-phosphorylated proteins were concurrently elevated or decreased. Taken together, these data suggest that blocking NKCC1 activity has impact on TMZ-induced activation of the WNK1-SPAK/OSR1-NKCC1 signaling pathway in both GL26 and SB28 glioma cells.

### STS66 Reduced Activation of AKT/ERK Signaling Pathway in Cultured Glioma Cells in Response to TMZ

**Figure 5:**
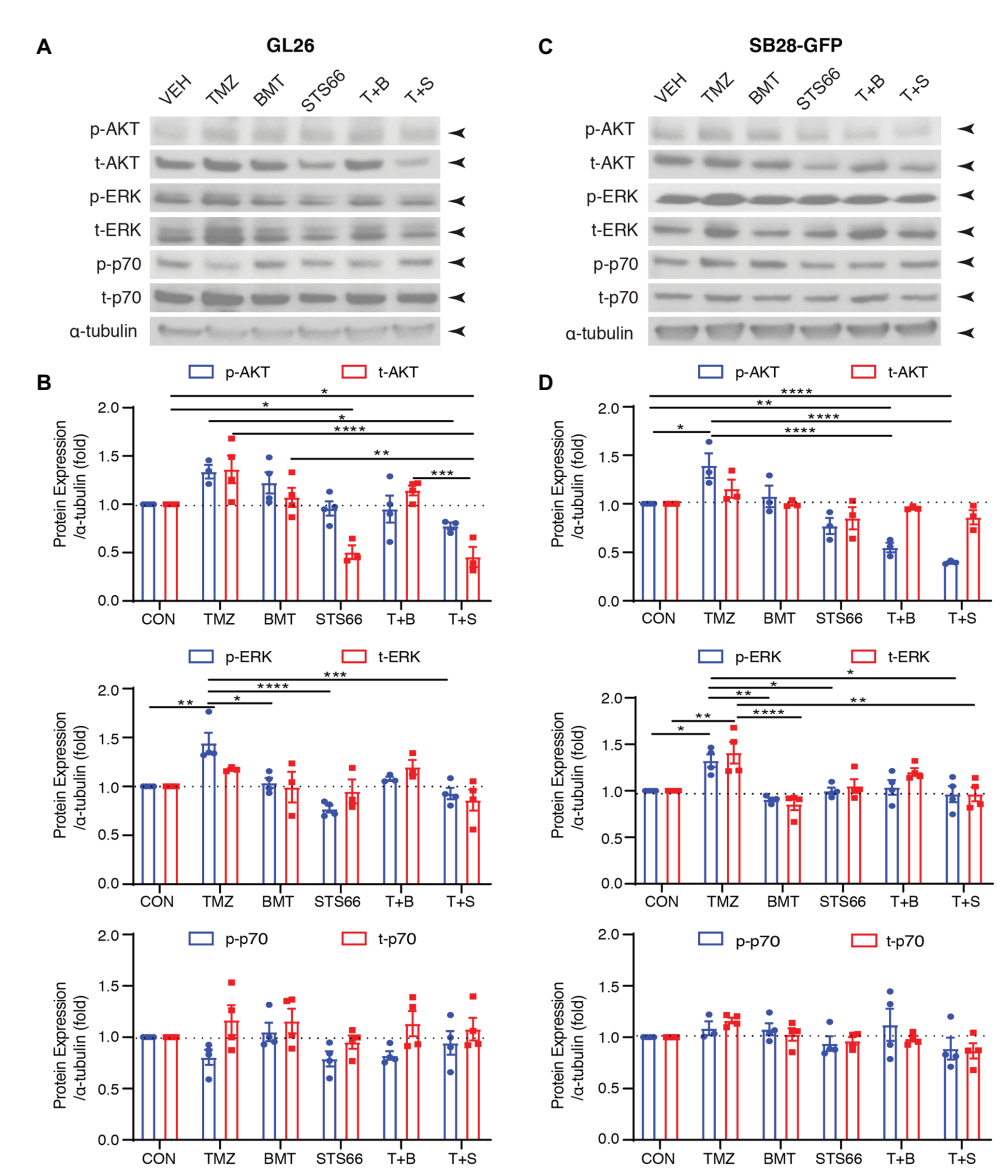
STS66 reduced activation of AKT and ERK but not mTOR protein in cultured glioma cells in response to TMZ. **(A)** Representative immunoblotting images of phosphorylated (p-) or total (t-) AKT, ERK, and p70 of the same cohorts of immunoblots in Figure 4. **(B)** Summary. Data are means ± SEM (*n* = 3, 4), ^*^p < 0.05, ^**^p < 0.01, ^***^p < 0.001, ^****^p < 0.0001. **(C)** Representative immunoblotting images of phosphorylated (p-) or total (t-) AKT, Erk and p70 of the same cohorts of immunoblots in Figure 4. **(D)** Summary. Data are means ± SEM (*n* = 3, 4), ^*^p < 0.05, ^**^p < 0.01, ^***^p < 0.001, ^****^p < 0.0001. All blots were re-stripped and re-probed from the same blots of Figure 4 and the original tubulin blots were used for normalization analysis.

AKT is involved in various cellular processes, including cell proliferation, survival, growth, and metabolism. AKT kinase activity can be activated in response to clinically relevant concentrations of TMZ. We subsequently probed changes of ERK/AKT activations and mTOR complex 1 (mTORC1) in the same sets of immunoblots of Figure 4. As shown in Figures 5A,B, exposing GL26 to TMZ for 48 h triggered an increase of t-AKT and p-AKT expression, but which did not reach statistical significance. TMZ caused ~44.2% higher p-ERK protein expression than Veh controls (*p* < 0.01, Figures 5A,B). However, no significant changes of mTORC1 expression, either non-phosphorylated (p70) or phosphorylated mTORC1 (p-p70), were detected in GL26 cells treated with TMZ (Figures 5A,B). Exposing GL26 to STS66 alone, but not NKCC1 inhibitor BMT, significantly downregulated t-AKT (*p* < 0.05) and inhibited ERK activation (*p* < 0.05). TMZ + STS66 treatment in GL26 cells also significantly inhibited TMZ-triggered AKT (p-AKT, *p* < 0.05) and ERK activation (p-ERK, *p* < 0.001; Figures 5A,B). Similar results were observed in SB28-GFP cells (Figures 5C,D). The ratios of p-/t-AKT, p-/t-ERK, and p-/t-p70 were presented in Supplementary Figure S3. It has shown that TMZ stimulates AKT and ERK pathways in human glioma cells (Sun et al., 2012; Bi et al., 2018) but formation of NKCC1 protein and Leu transporter LAT1 complex inhibits AKT/ERK-mTOR1 activation in epithelial cells in response to amino acid-mediated stimulation (Demian et al., 2019). Our data demonstrate that inhibition of NKCC1 protein in glioma cells with BMT or STS66 suppresses AKT and ERK signaling pathways in response to TMZ stress but has no effects on mTORC1 signaling. The discrepancy could be due to different cellular responses to different stimuli.

## Discussion

### Significant Role of NKCC1 Protein in Glioma Ionic Regulation

NKCC1 protein is a Na^+^-dependent Cl^−^ transporter that maintaining intracellular K^+^ and Cl^−^ concentration and cell volume homeostasis by regulating the movement of Na^+^, K^+^, and Cl^−^ across the plasma membrane (Cong et al., 2015). A growing body of evidence indicates that expression of NKCC1 protein is closely related to tumor progression, such as lung adenocarcinoma (Sun et al., 2016), prostate cancer cells (Hiraoka et al., 2010), and esophageal squamous carcinoma cells (Shiozaki et al., 2014). Luo et al. showed that NKCC1 protein and messenger RNA (mRNA) are abundantly expressed in all grade of gliomas (Luo et al., 2020). Unlike other cancers that spread through the vasculature, gliomas actively invade surrounding brain solely along extracellular routes (Watkins and Sontheimer, 2011). Invasion of glioma cells starts at narrow extracellular space through the activities of the ion channels to carry out the cell shape and volume changes (McFerrin and Sontheimer, 2006). Therefore, glioma cells need to establish an appropriate ion gradient to facilitate cell migration, and NKCC1 protein which carries Na^+^, K^+^, and 2Cl^−^ into cells contributes to establishing the ion gradient for intracellular K^+^ and Cl^−^ efflux (Cuddapah and Sontheimer, 2011). In our study, we reported here that NKCC1 protein activity constitutes an important K^+^ (Rb^+^) influx mechanism in mouse glioma GL26 cells (~60% of total Rb^+^ influx) and SB28-GFP cells (~20% of total Rb^+^ influx) either under basal homeostatic conditions or in response to osmotic stress-induced cell shrinkage. Both the well-established NKCC1 inhibitor BMT and the new BMT-derivative STS66 are able to block NKCC1 activity and Rb^+^ influx. Our findings show that STS66 is more effective than BMT in inhibiting the activation of NKCC1 protein and reduced NKCC1-mediated Rb^+^ influx, which hindered the establishment of intracellular ion gradient in cultured glioma cells.

### NKCC1 Protein Activation in Glioma K^+^ (Rb^+^) Influx and Proliferation in Response to TMZ-Induced Apoptosis

Among the earliest events that occur after mitogenic stimulation in the early G0/G1-phase of the cell cycle is an increase of K^+^ influx (Panet et al., 2000) and cell volume increase of proliferating cells before they enter the S-phase (Habela et al., 2009). The asymmetrical distribution of Na^+^ and K^+^ is characteristic of many animal cells and the high intracellular concentration of K^+^ is necessary for the transition of quiescence to proliferating state of human peripheral blood cells (Marakhova et al., 2019). NKCC1 activity was shown to be essential to enter the S-phase of cell cycle in Balb/c 3T3 fibroblast cells (Panet et al., 2000). Additionally, high intracellular Cl^−^ level serves as an osmolyte and energetic driving force for volume changes and is important for cellular proliferation. High intracellular Cl^−^ level (~100 mM) is maintained in glioma cells through the activity of NKCC1 (Turner and Sontheimer, 2014). Previously, we have shown that glioma NKCC1 plays an important role in intracellular K^+^, Cl^−^ accumulation, and RVI in response to osmotic stress or AVD (Algharabil et al., 2012). In this study, 48 h exposure of TMZ increased NKCC1 activity (increased K^+^ influx) in GL26 and SB28-GFP glioma cells. In contrast, glioma cells treated with STS66 alone or in combination with TMZ reduced the NKCC1 activity. TMZ causes methylation of DNA resulting in DNA fragmentation and disruption of replication known to induce cell cycle arrest in G2/M phase leading to apoptosis (Lee, 2016). In the current study, at 24 h post TMZ treatment, the proliferation of GL26 and SB28-GFP glioma cells was not significantly reduced in the presence or absence of BMT, which is consistent with previous findings (Algharabil et al., 2012). However, 48 h of TMZ exposure reduced GL26 cell proliferation. It requires high dose of TMZ or longer exposure to affect cellular proliferation. The IC50 value of TMZ at 24 h exposure was 5 mM in SH-SY5Y neuroblastoma cells (Çıtışlı et al., 2015) and at concentration of 25–50 μM, it took 72 h to significantly reduce the proliferation of LN229 and U87 glioma cells (Aasland et al., 2019). Our results are in line with these reports that TMZ works in a time-dependent manner to affect cellular proliferation. In contrast, treatment of glioma cells with STS66 (60 μM) alone or TMZ plus STS66 for 24 or 48 h significantly reduced glioma cells proliferation and induced G0/G1 arrest, which appears to correlate with NKCC1-mediated reduced K^+^ influx. Consistently, a recent report showed that proliferating cells had increased number of cells in G0/G1 phase (Demian et al., 2019). In summary, in this study, blocking NKCC1 activity with STS66 significantly reduced glioma cells proliferation, which is likely due to reduced intracellular K^+^ and Cl^−^ concentration and prevents cell volume increase during G1 to S-phase of the cell cycle and reduces proliferation.

### Differences Between BMT and STS66

According to our Rb^+^ influx assay, STS66 requires a higher concentration (60 μM) than BMT (10 μM) to block the NKCC1-mediated Rb^+^ influx, which indicates that STS66 has a higher IC50 value than BMT probably due to the structural differences. In STS66, the carboxylic group of BMT was exchanged with a trifluoroethylaminomethyl group, vastly increasing its lipophilicity (Huang et al., 2019; Auer et al., 2020b). On the other hand, this structural difference may improve blood-brain barrier (BBB) penetration by increasing the molecule’s lipophilicity caused by the exchange for the amine partial structure. Our recent study showed that under the same molar concentration of drugs, STS66 (12 mg/kg mouse weight/day) is more effective than BMT (10 mg/kg mouse weight/day) in reducing infarction and improving mouse outcomes after ischemic stroke (Huang et al., 2019). In this current study, we found that STS66 as mono-treatment or combined with chemotherapeutic drug TMZ, reduces glioma cell proliferation. Further studies are needed to investigate efficacy of STS66 in anti-tumor *in vivo* experiments. Considering the diuretic actions of BMT (Younus and Reddy, 2018) and its hearing impairment at high dose (Allegaert et al., 2016), the new BMT-derivative drugs such as STS66 may be more effective in blocking brain NKCC1 protein activity with less side effects.

A new study in which part of the authors were involved showed improved efficacy of compounds similar to STS66 in treating epilepsy compared to BMT itself (Auer et al., 2020a). No diuretic effects were observed at any dose of these derivatives. This shows therapeutic potentials for the improved BMT derivatives in many applications and makes this particular area especially interesting for further research.

### Impact of NKCC1 Blockade on K^+^ Flux, WNK1-SPAK-NKCC1 Complex, and AKT/ERK Signaling in Glioma Cells

In addition to inhibition of K^+^ influx, STS66 (60 μM) significantly reduced the proliferation of both GL26 and SB28 cells. The maximum inhibition effects were obtained with TMZ + STS66 (60 μM) combination. The same trend was also found by cell cycle analysis. These likely are direct impact of blocking NKCC1 protein activity on cell proliferation and cell cycle. However, we cannot rule out that blocking NKCC1 protein activity may indirectly affect other proteins. In Figure 4, BMT, STS66 (10 μM) and the combined treatment of TMZ + BMT or TMZ + STS66 downregulated WNK1 protein expression. In Figure 5, STS66 (10 μM) in combination with TMZ significantly inhibited AKT or ERK activation but not mTOR signaling. The mechanisms underlying these changes are not clear. It may indirectly mediate through changes of cell volume or other signaling pathways, such as ERK/AKT. AKT is involved in various cellular processes, including cell proliferation, survival, growth, and metabolism. AKT kinase activity can be activated in response to clinically relevant concentrations of TMZ. These findings suggest that blockade of NKCC1 protein activity may indirectly affect protein expression *via* changing cell volume or ERK/AKT pathways. The detailed underlying mechanisms require further investigation in the future.

## Data Availability Statement

All datasets presented in this study are included in the article/Supplementary Material.

## Author Contributions

LL, JW, DD, S-SY, S-HL, PS, TE, BS, YY, and DS designed the experiments. LL, JW, and DD performed K^+^ influx, proliferation, and immunoblotting assay. NH performed MTT and cell cycle assay. LL, JW, NH, TE, and DS wrote, proofread, and edited the manuscript. All authors contributed to the article and approved the submitted version.

### Conflict of Interest

The authors declare that the research was conducted in the absence of any commercial or financial relationships that could be construed as a potential conflict of interest.

## Supplementary Material

Supplementary Figure S1 STS66 reduces glioma bromodeoxyuridine (BrdU) incorporation in combination of chemoreagent temozolomide. **(A,B)** Relative cell proliferation of BrdU incorporation assay. GL26 or SB28-GFP cells were exposed to DMSO (Veh), TMZ (100 μM), BMT (10 μM), STS66 (60 μM), T + B, or T + S combined for 48 h. Data are means ± SEM (*n* = 3), ^*^*p* < 0.05, ^**^*p* < 0.01, ^***^*p* < 0.001, ^****^*p* < 0.0001.

Supplementary Figure S2STS66 reduced activation of WNK1-SPAK/OSR1-NKCC1 cascade in cultured glioma cells in response to temozolomide (TMZ). **(A–F)** Summary data of the ratios of phosphorylated (p-)/total (t-) WNK1, SPAK/OSR1, and NKCC1 protein. Data are means ± SEM (*n* = 4, 5), ^*^*p* < 0.05, ^**^*p* < 0.01, ^***^*p* < 0.001.

Supplementary Figure S3STS66 reduced activation of AKT and ERK but not mTOR protein in cultured glioma cells in response to TMZ. **(A–F)** Summary data of the ratios of phosphorylated (p-)/total (t-) AKT, ERK, and p70 protein. Data are means ± SEM (*n* = 3, 4), ^*^*p* < 0.05, ^**^*p* < 0.01, ^***^*p* < 0.001.

## References

[ref1] Aasland D. Götzinger L. Hauck L. Berte N. Meyer J. Effenberger M.. (2019). Temozolomide induces senescence and repression of DNA repair pathways in glioblastoma cells via activation of ATR-CHK1, p21, and NF-κB. Cancer Res. 79, 99–113. 10.1158/0008-5472.CAN-18-1733, PMID: 30361254

[ref2] Algharabil J. Kintner D. B. Wang Q. Begum G. Clark P. A. Yang S. S.. (2012). Inhibition of Na^+^-K^+^-2Cl^−^ cotransporter isoform 1 accelerates temozolomide-mediated apoptosis in glioblastoma cancer cells. Cell. Physiol. Biochem. 30, 33–48. 10.1159/000339047, PMID: 22759954 PMC3603147

[ref3] Allegaert K. Lahav A. van den Anker J. N. (2016). Erratum to: a mechanism to explain ototoxicity in neonates exposed to bumetanide: lessons to help improve future product development in neonates. Paediatr. Drugs 18:475. 10.1007/s40272-016-0195-z, PMID: 27665286

[ref4] Aronica E. Boer K. Redeker S. Spliet W. G. van Rijen P. C. Troost D.. (2007). Differential expression patterns of chloride transporters, Na^+^-K^+^-2Cl^−^-cotransporter and K^+^-Cl^−^-cotransporter, in epilepsy-associated malformations of cortical development. Neuroscience 145, 185–196. 10.1016/j.neuroscience.2006.11.041, PMID: 17207578

[ref5] Auer T. Schreppel P. Erker T. Schwarzer C. (2020a). Functional characterization of novel bumetanide derivatives for epilepsy treatment. Neuropharmacology 162:107754. 10.1016/j.neuropharm.2019.107754, PMID: 31476353

[ref6] Auer T. Schreppel P. Erker T. Schwarzer C. (2020b). Impaired chloride homeostasis in epilepsy: molecular basis, impact on treatment, and current treatment approaches. Pharmacol. Ther. 205:107422. 10.1016/j.pharmthera.2019.107422, PMID: 31626872

[ref7] Bi Y. Li H. Yi D. Sun Y. Bai Y. Zhong S.. (2018). Cordycepin augments the chemosensitivity of human glioma cells to temozolomide by activating AMPK and inhibiting the AKT signaling pathway. Mol. Pharm. 15, 4912–4925. 10.1021/acs.molpharmaceut.8b00551, PMID: 30336060

[ref8] Bortner C. D. Cidlowski J. A. (2007). Cell shrinkage and monovalent cation fluxes: role in apoptosis. Arch. Biochem. Biophys. 462, 176–188. 10.1016/j.abb.2007.01.020, PMID: 17321483 PMC1941616

[ref9] Brandt C. Seja P. Töllner K. Römermann K. Hampel P. Kalesse M.. (2018). Bumepamine, a brain-permeant benzylamine derivative of bumetanide, does not inhibit NKCC1 but is more potent to enhance phenobarbital’s anti-seizure efficacy. Neuropharmacology 143, 186–204. 10.1016/j.neuropharm.2018.09.025, PMID: 30248303

[ref10] Çıtışlı V. Dodurga Y. Eroğlu C. Seçme M. Avcı Ç. B. Şatıroğlu-Tufan N. L. (2015). Temozolomide may induce cell cycle arrest by interacting with URG4/URGCP in SH-SY5Y neuroblastoma cells. Tumour Biol. 36, 6765–6772. 10.1007/s13277-015-3373-7, PMID: 25835972

[ref11] Cong D. Zhu W. Kuo J. S. Hu S. Sun D. (2015). Ion transporters in brain tumors. Curr. Med. Chem. 22, 1171–1181. 10.2174/0929867322666150114151946, PMID: 25620102 PMC4363268

[ref12] Cuddapah V. A. Sontheimer H. (2011). Ion channels and transporters [corrected] in cancer. 2. Ion channels and the control of cancer cell migration. Am. J. Physiol. Cell Physiol. 301, C541–C549. 10.1152/ajpcell.00102.2011, PMID: 21543740 PMC3174565

[ref13] Delpire E. Gagnon K. B. (2018). Water homeostasis and cell volume maintenance and regulation. Curr. Top. Membr. 81, 3–52. 10.1016/bs.ctm.2018.08.001, PMID: 30243436 PMC6457474

[ref14] Demian W. L. Persaud A. Jiang C. Coyaud É. Liu S. Kapus A.. (2019). The ion transporter NKCC1 links cell volume to cell mass regulation by suppressing mTORC1. Cell Rep. 27, 1886–1896.E6. 10.1016/j.celrep.2019.04.034, PMID: 31067471

[ref15] Gamba G. (2005). Molecular physiology and pathophysiology of electroneutral cation-chloride cotransporters. Physiol. Rev. 85, 423–493. 10.1152/physrev.00011.2004, PMID: 15788703

[ref16] Garzon-Muvdi T. Schiapparelli P. ap Rhys C. Guerrero-Cazares H. Smith C. Kim D. H.. (2012). Regulation of brain tumor dispersal by NKCC1 through a novel role in focal adhesion regulation. PLoS Biol. 10:e1001320. 10.1371/journal.pbio.1001320, PMID: 22570591 PMC3341330

[ref17] Haas B. R. Cuddapah V. A. Watkins S. Rohn K. J. Dy T. E. Sontheimer H. (2011). With-no-lysine kinase 3 (WNK3) stimulates glioma invasion by regulating cell volume. Am. J. Physiol. Cell Physiol. 301, C1150–C1160. 10.1152/ajpcell.00203.2011, PMID: 21813709 PMC3213919

[ref18] Haas B. R. Sontheimer H. (2010). Inhibition of the sodium-potassium-chloride cotransporter isoform-1 reduces glioma invasion. Cancer Res. 70, 5597–5606. 10.1158/0008-5472.CAN-09-4666, PMID: 20570904 PMC2896443

[ref19] Habela C. W. Ernest N. J. Swindall A. F. Sontheimer H. (2009). Chloride accumulation drives volume dynamics underlying cell proliferation and migration. J. Neurophysiol. 101, 750–757. 10.1152/jn.90840.2008, PMID: 19036868 PMC2657062

[ref20] Hiraoka K. Miyazaki H. Niisato N. Iwasaki Y. Kawauchi A. Miki T.. (2010). Chloride ion modulates cell proliferation of human androgen-independent prostatic cancer cell. Cell. Physiol. Biochem. 25, 379–388. 10.1159/000303042, PMID: 20332618

[ref21] Hoffmann E. K. Lambert I. H. (2014). Ion channels and transporters in the development of drug resistance in cancer cells. Philos. Trans. R. Soc. Lond. B Biol. Sci. 369:20130109. 10.1098/rstb.2013.0109, PMID: 24493757 PMC3917363

[ref22] Hoffmann E. K. Lambert I. H. Pedersen S. F. (2009). Physiology of cell volume regulation in vertebrates. Physiol. Rev. 89, 193–277. 10.1152/physrev.00037.2007, PMID: 19126758

[ref23] Huang H. Bhuiyan M. I. H. Jiang T. Song S. Shankar S. Taheri T.. (2019). A novel Na^+^-K^+^-Cl^−^ cotransporter 1 inhibitor STS66* reduces brain damage in mice after ischemic stroke. Stroke 50, 1021–1025. 10.1161/STROKEAHA.118.024287, PMID: 30862257 PMC6608592

[ref24] Hughes F. M. Jr. Cidlowski J. A. (1999). Potassium is a critical regulator of apoptotic enzymes in vitro and in vivo. Adv. Enzyme Regul. 39, 157–171. 10.1016/S0065-2571(98)00010-7, PMID: 10470372

[ref25] Kohanbash G. Carrera D. A. Shrivastav S. Ahn B. J. Jahan N. Mazor T.. (2017). Isocitrate dehydrogenase mutations suppress STAT1 and CD8^+^ T cell accumulation in gliomas. J. Clin. Invest. 127, 1425–1437. 10.1172/JCI90644, PMID: 28319047 PMC5373859

[ref26] Lee S. Y. (2016). Temozolomide resistance in glioblastoma multiforme. Genes Dis. 3, 198–210. 10.1016/j.gendis.2016.04.007, PMID: 30258889 PMC6150109

[ref27] Luo L. Guan X. Begum G. Ding D. Gayden J. Hasan M. N.. (2020). Blockade of cell volume regulatory protein NKCC1 increases TMZ-induced glioma apoptosis and reduces astrogliosis. Mol. Cancer Ther. 19, 1550–1561. 10.1158/1535-7163.MCT-19-0910, PMID: 32393472 PMC11792748

[ref28] Maeno E. Takahashi N. Okada Y. (2006). Dysfunction of regulatory volume increase is a key component of apoptosis. FEBS Lett. 580, 6513–6517. 10.1016/j.febslet.2006.10.074, PMID: 17101138

[ref29] Marakhova I. Yurinskaya V. Aksenov N. Zenin V. Shatrova A. Vereninov A. (2019). Intracellular K^+^ and water content in human blood lymphocytes during transition from quiescence to proliferation. Sci. Rep. 9:16253. 10.1038/s41598-019-52571-1 31700012 PMC6838062

[ref30] McFerrin M. B. Sontheimer H. (2006). A role for ion channels in glioma cell invasion. Neuron Glia Biol. 2, 39–49. 10.1017/S1740925X06000044, PMID: 16520829 PMC1389710

[ref31] Moriguchi T. Urushiyama S. Hisamoto N. Iemura S. I. Uchida S. Natsume T.. (2005). WNK1 regulates phosphorylation of cation-chloride-coupled cotransporters via the STE20-related kinases, SPAK and OSR1. J. Biol. Chem. 280, 42685–42693. 10.1074/jbc.M510042200, PMID: 16263722

[ref32] Okada Y. Maeno E. (2001). Apoptosis, cell volume regulation and volume-regulatory chloride channels. Comp. Biochem. Physiol. A Mol. Integr. Physiol. 130, 377–383. 10.1016/S1095-6433(01)00424-X, PMID: 11913451

[ref33] Panet R. Marcus M. Atlan H. (2000). Overexpression of the Na^+^/K^+^/Cl^−^ cotransporter gene induces cell proliferation and phenotypic transformation in mouse fibroblasts. J. Cell. Physiol. 182, 109–118. 10.1002/(SICI)1097-4652(200001)182:1<109::AID-JCP12>3.0.CO;2-A, PMID: 10567922

[ref34] Poulsen K. A. Andersen E. C. Hansen C. F. Klausen T. K. Hougaard C. Lambert I. H.. (2010). Deregulation of apoptotic volume decrease and ionic movements in multidrug-resistant tumor cells: role of chloride channels. Am. J. Physiol. Cell Physiol. 298, C14–C25. 10.1152/ajpcell.00654.2008, PMID: 19846756

[ref35] Sareddy G. R. Li X. Liu J. Viswanadhapalli S. Garcia L. Gruslova A.. (2016). Selective estrogen receptor β agonist LY500307 as a novel therapeutic agent for glioblastoma. Sci. Rep. 6:24185. 10.1038/srep24185, PMID: 27126081 PMC4850367

[ref36] Schiapparelli P. Guerrero-Cazares H. Magaña-Maldonado R. Hamilla S. M. Ganaha S. Goulin Lippi Fernandes E.. (2017). NKCC1 regulates migration ability of glioblastoma cells by modulation of actin dynamics and interacting with cofilin. EBioMedicine 21, 94–103. 10.1016/j.ebiom.2017.06.020, PMID: 28679472 PMC5514434

[ref37] Shiozaki A. Nako Y. Ichikawa D. Konishi H. Komatsu S. Kubota T.. (2014). Role of the Na^+^/K^+^/2Cl(^−^) cotransporter NKCC1 in cell cycle progression in human esophageal squamous cell carcinoma. World J. Gastroenterol. 20, 6844–6859. 10.3748/wjg.v20.i22.6844, PMID: 24944475 PMC4051924

[ref38] Skou J. C. Esmann M. (1992). The Na,K-ATPase. J. Bioenerg. Biomembr. 24, 249–261. 10.1007/BF00768846, PMID: 1328174

[ref39] Sun P. L. Jin Y. Park S. Y. Kim H. Park E. Jheon S.. (2016). Expression of Na^+^-K^+^-2Cl^−^ cotransporter isoform 1 (NKCC1) predicts poor prognosis in lung adenocarcinoma and EGFR-mutated adenocarcinoma patients. QJM 109, 237–244. 10.1093/qjmed/hcv207, PMID: 26559081

[ref40] Sun S. Lee D. Lee N. P. Pu J. K. Wong S. T. Lui W. M.. (2012). Hyperoxia resensitizes chemoresistant human glioblastoma cells to temozolomide. J. Neurooncol. 109, 467–475. 10.1007/s11060-012-0923-3, PMID: 22763762 PMC3434886

[ref41] Thier S. O. (1986). Potassium physiology. Am. J. Med. 80, 3–7. 10.1016/0002-9343(86)90334-7, PMID: 3706350

[ref42] Töllner K. Brandt C. Töpfer M. Brunhofer G. Erker T. Gabriel M.. (2014). A novel prodrug-based strategy to increase effects of bumetanide in epilepsy. Ann. Neurol. 75, 550–562. 10.1002/ana.24124, PMID: 24615913

[ref43] Turner K. L. Sontheimer H. (2014). Cl^−^ and K^+^ channels and their role in primary brain tumour biology. Philos. Trans. R. Soc. Lond. B Biol. Sci. 369:20130095. 10.1098/rstb.2013.0095, PMID: 24493743 PMC3917349

[ref44] Watkins S. Sontheimer H. (2011). Hydrodynamic cellular volume changes enable glioma cell invasion. J. Neurosci. 31, 17250–17259. 10.1523/JNEUROSCI.3938-11.2011, PMID: 22114291 PMC3253353

[ref45] Wehner F. Olsen H. Tinel H. Kinne-Saffran E. Kinne R. K. (2003). Cell volume regulation: osmolytes, osmolyte transport, and signal transduction. Rev. Physiol. Biochem. Pharmacol. 148, 1–80. 10.1007/s10254-003-0009-x, PMID: 12687402

[ref46] Yang S. S. Lo Y. F. Wu C. C. Lin S. W. Yeh C. J. Chu P. L.. (2010). SPAK-knockout mice manifest gitelman syndrome and impaired vasoconstriction. J. Am. Soc. Nephrol. 21, 1868–1877. 10.1681/ASN.2009121295, PMID: 20813865 PMC3014002

[ref47] Younus I. Reddy D. S. (2018). A resurging boom in new drugs for epilepsy and brain disorders. Expert Rev. Clin. Pharmacol. 11, 27–45. 10.1080/17512433.2018.1386553, PMID: 28956955

[ref48] Yu S. P. (2003). Regulation and critical role of potassium homeostasis in apoptosis. Prog. Neurobiol. 70, 363–386. 10.1016/S0301-0082(03)00090-X, PMID: 12963093

[ref49] Zhu W. Begum G. Pointer K. Clark P. A. Yang S. S. Lin S. H.. (2014). WNK1-OSR1 kinase-mediated phospho-activation of Na^+^-K^+^-2Cl^−^ cotransporter facilitates glioma migration. Mol. Cancer 13:31. 10.1186/1476-4598-13-31, PMID: 24555568 PMC3936893

