# Supplementary figures and images for: Role of NKCC1 Activity in Glioma K+ Homeostasis and Cell Growth: New Insights With the Bumetanide-Derivative STS66
Source: Front Physiol. 2020 Jul 31;11:911. doi: 10.3389/fphys.2020.00911 (PMC7413028; doi:10.3389/fphys.2020.00911)

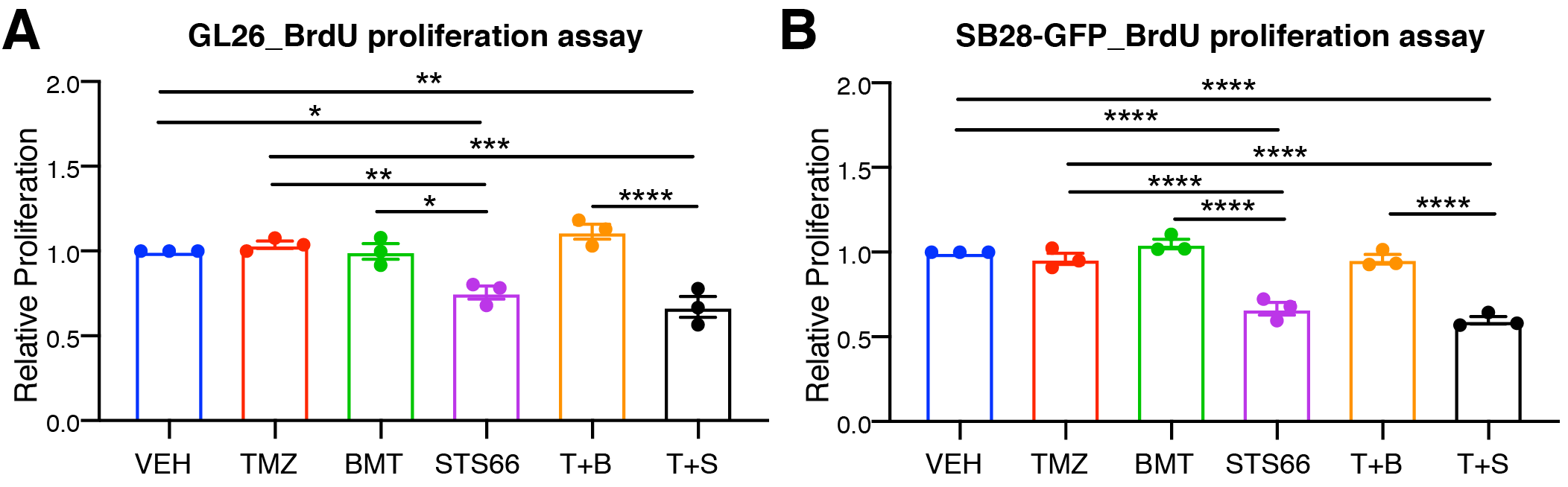

Supplement: Supplementary file 1 [file Image_1.PNG]

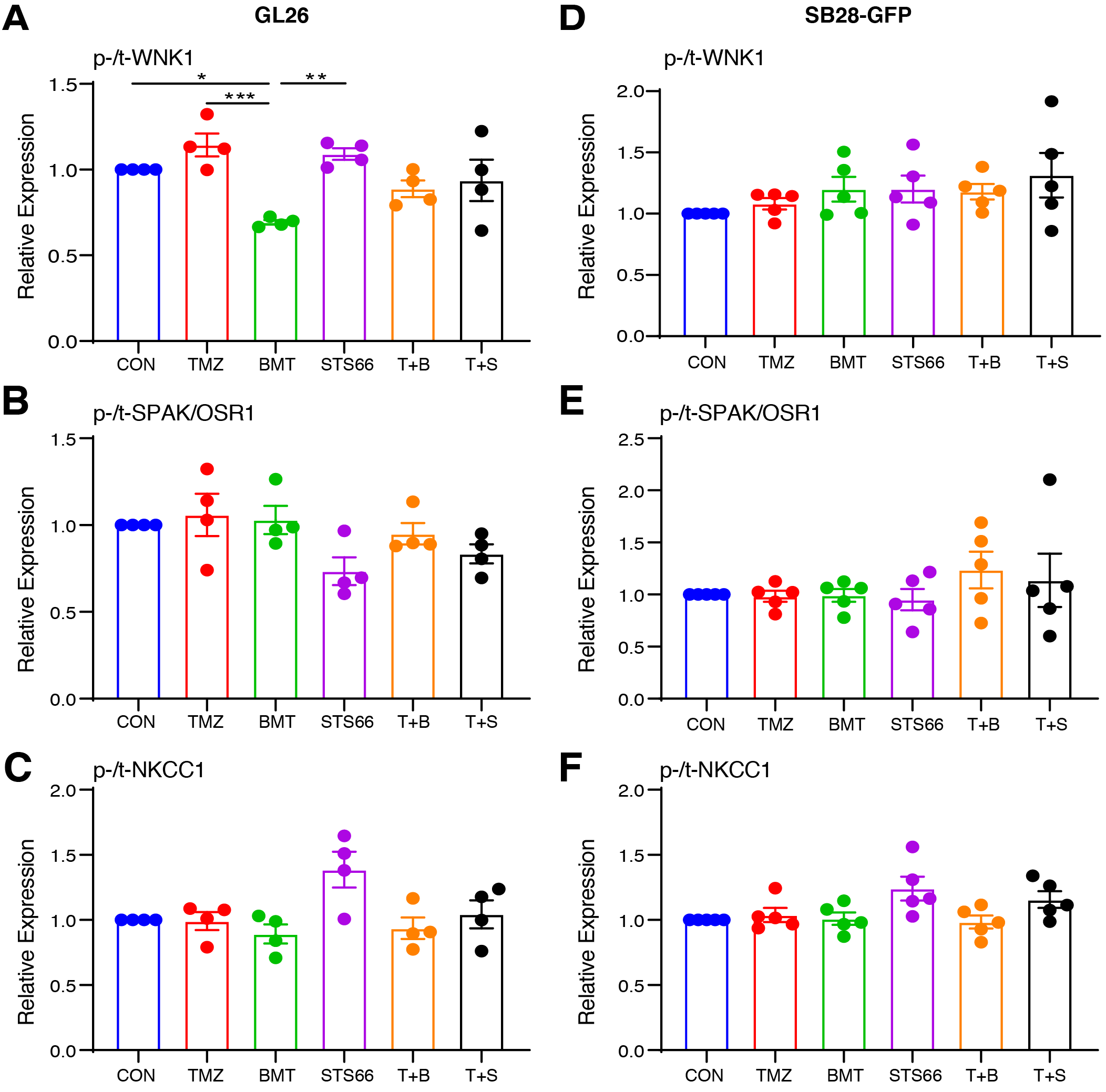

Supplement: Supplementary file 2 [file Image_2.PNG]

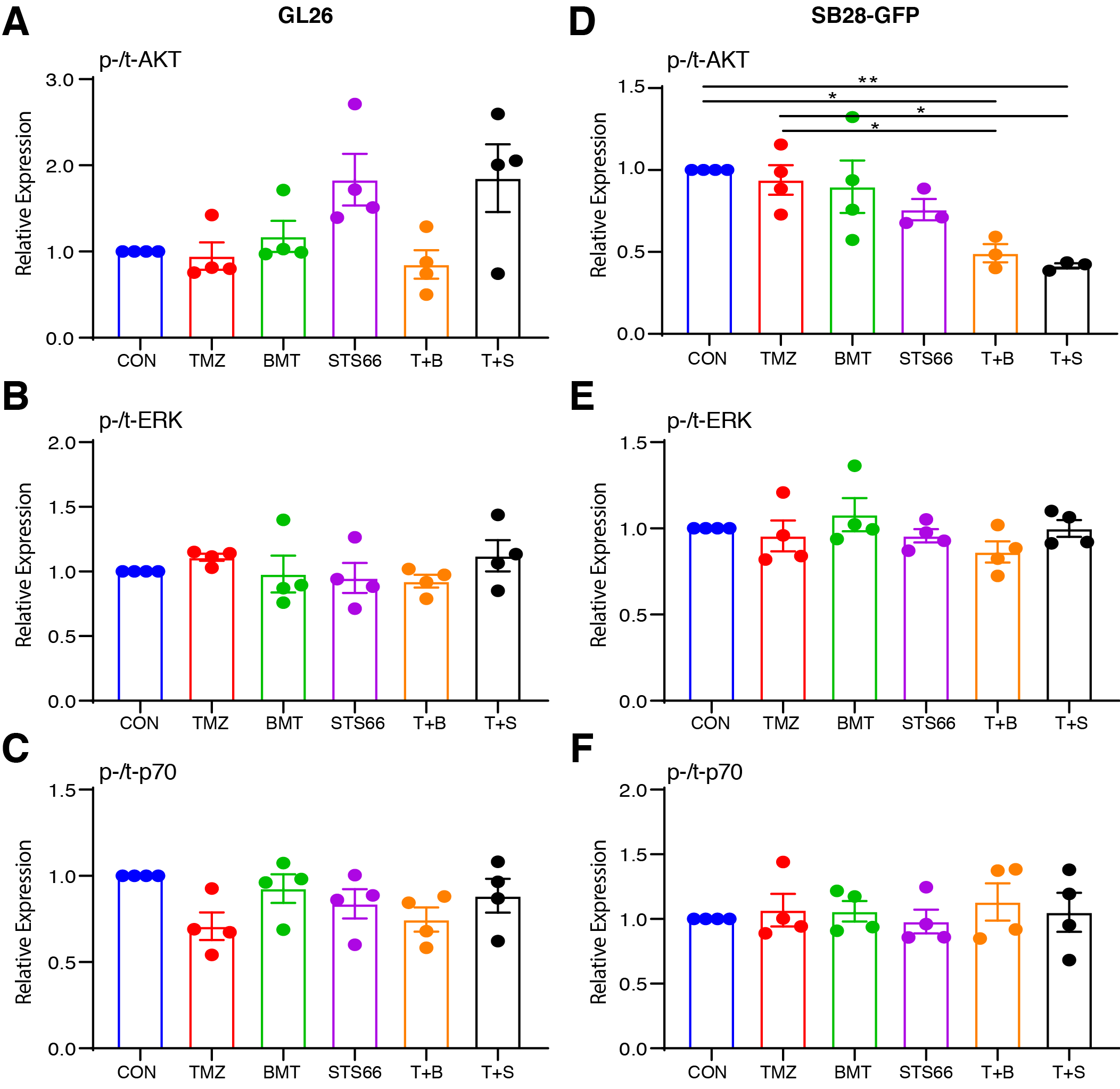

Supplement: Supplementary file 3 [file Image_3.PNG]
